# Supplementary material for: The causal relationship between the human gut microbiota and pyogenic arthritis: a Mendelian randomization study
Source: Front Cell Infect Microbiol. 2024 Nov 26;14:1452480. doi: 10.3389/fcimb.2024.1452480 (PMC11629706; doi:10.3389/fcimb.2024.1452480)
Supplement: Supplementary file 1 [file DataSheet1.zip › mendelian test/ebi-a-GCST90027585.csv_leave_one_out.pdf]

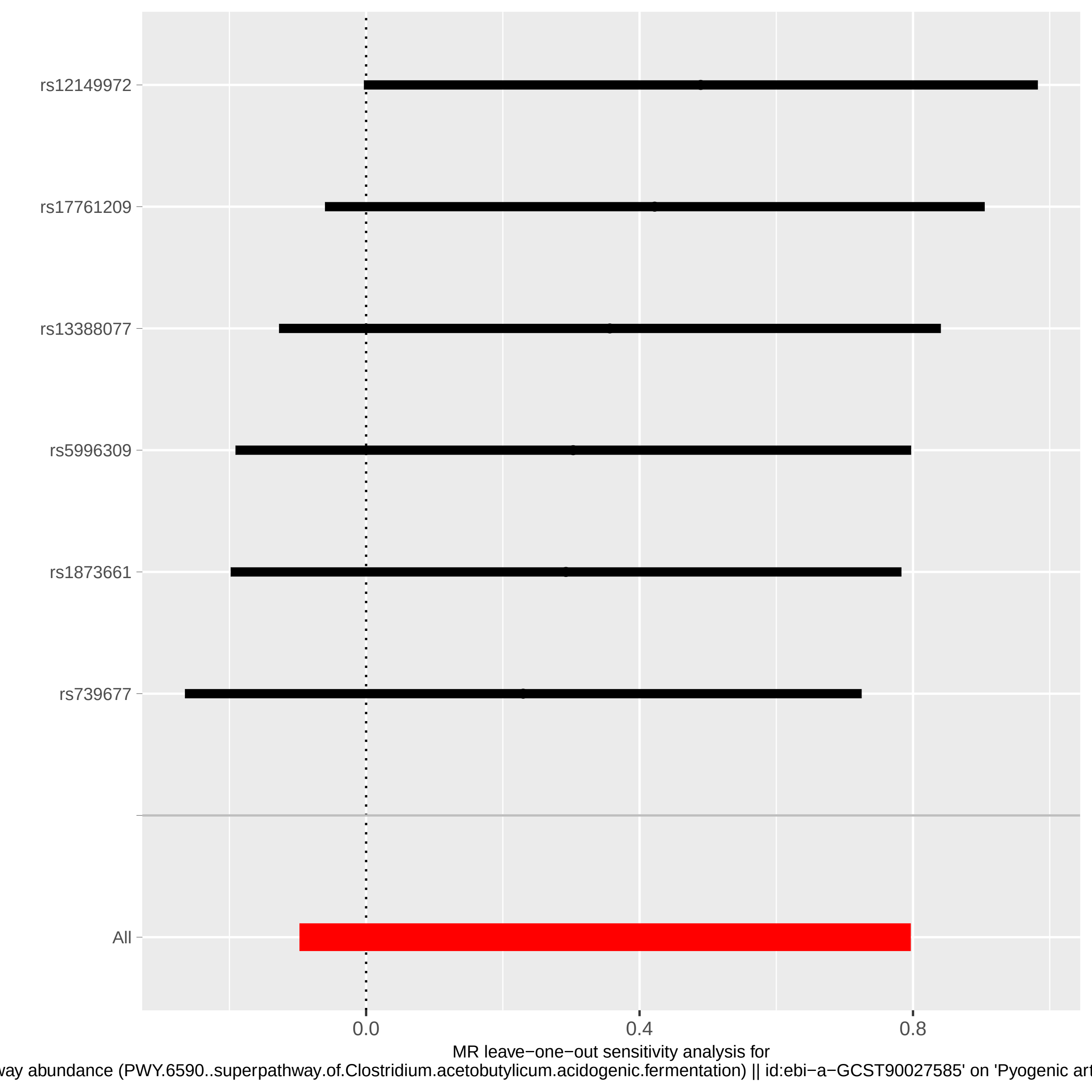

rs12149972
rs17761209
rs13388077
rs5996309
rs1873661
rs739677
All
0.0
0.4
0.8
MR leave−one−out sensitivity analysis for
way abundance (PWY.6590..superpathway.of.Clostridium.acetobutylicum.acidogenic.fermentation) || id:ebi−a−GCST90027585' on 'Pyogenic art
